# Depression in relation to sex and gender expression among Swedish septuagenarians—Results from the H70 study

**DOI:** 10.1371/journal.pone.0238701

**Published:** 2020-09-14

**Authors:** Therese Rydberg Sterner, Pia Gudmundsson, Hanna Falk, Nazib Seidu, Felicia Ahlner, Hanna Wetterberg, Lina Rydén, Robert Sigström, Svante Östling, Anna Zettergren, Silke Kern, Margda Waern, Ingmar Skoog

**Affiliations:** 1 Department of Psychiatry and Neurochemistry, Neuropsychiatric Epidemiology Unit, Institute of Neuroscience and Physiology, Sahlgrenska Academy, Centre for Ageing and Health (AGECAP) at the University of Gothenburg, Mölndal, Sweden; 2 Region Västra Götaland, Sahlgrenska University Hospital, Psychiatry, Cognition and Old Age Psychiatry Clinic, Gothenburg, Sweden; 3 Region Västra Götaland, Sahlgrenska University Hospital, Psychosis Clinic, Gothenburg, Sweden; Washington State University, UNITED STATES

## Abstract

**Objective:**

Little is known about the role of gender expression (femininity, masculinity, or androgyny) in relation to sex differences in depression. This study tested if gender expression was associated with depression and burden of depressive symptoms in a 70-year-old population.

**Methods:**

A cross-sectional population-based sample of 70-year-olds from The Gothenburg H70 Birth Cohort Study (n = 1203) was examined in 2014–16. Data were collected using psychiatric examinations and structured questionnaires, including the Positive-Negative Sex-Role Inventory to assess gender expression. Depression was diagnosed according to the Diagnostic and Statistical Manual of Mental Disorders criteria, and symptom burden was assessed with Montgomery Åsberg Depression Rating Scale (MADRS).

**Results:**

Gender expression was related to MADRS score and depression diagnosis. In fully adjusted models, feminine traits with low social desirability (FEM-) were associated with a higher MADRS score (R^2^ 0.16; B 0.16; CI 0.1–0.2), while androgyny (t ratio) (R^2^ 0.12; B 0.42; CI 0.1–0.7) and masculine traits with high social desirability (MAS+) (R^2^ 0.13; B -0.06; CI -0.1–-0.01) were associated with a lower MADRS score. Also, feminine traits with low social desirability (FEM-) were positively associated with depression (OR 1.04; CI 1.01–1.1). No associations between depression and masculinity or androgyny were observed in adjusted models. There were no interactions between sex and gender expression in relation to depression or MADRS score, indicating that the effects of gender expression were similar in men and women.

**Conclusions:**

We found that gender expression was associated to both depression and burden of depressive symptoms. More specifically, we found that femininity was associated to higher levels of depression, irrespective of biological sex. In addition, masculinity and androgyny were associated with lower levels of depression. These results highlight the importance of taking gender expression into consideration when studying sex differences in depression among older populations in future studies.

## Introduction

The Lancet series ‘Gender Equality, Norms, and Health’ [1] highlights the importance of treating sex and gender as distinct, not interchangeable, entities in medical research. While sex includes the biological distinction between men and women, gender adds to the behavioral, cultural or psychological attributes associated with one sex or the other according to societal norms, which can change over time and differ among cultures [2]. The theoretical framework of this paper is founded in the extended version of the sex role theory, in which the concept of psychological androgyny was launched in 1974 [3]. Within this theory, the term gender expression constitutes how an individual expresses a sense of being masculine, feminine, neither, or both through behavioral attributes. A person’s gender expression may not be fixed by nature, nor solely imposed from societal norms [4]. However, gender expression is suggested to include actively learning and incorporating gendered patterns into the self-concept, across the life course [4]. Even though masculinity is stereotypically associated to men, and femininity to women, these characteristics are present, to differing degrees, in most persons [4]. With this in mind, masculinity and femininity are not on opposite sides of a continuum. Instead, they compose a two-dimensional construct where masculinity and femininity comprise one dimension each [5]. There is an ongoing scientific discussion whether femininity, masculinity and androgyny should be termed ‘gender identity’, ‘gender role orientation’, ‘sex role’ or ‘gender expression’. This discussion generates discrepancies among studies. However, based on theoretical discussions published in the 2019 Lancet series ‘Gender equality, norms and health’ [1], ‘gender expression’ is used within this paper when discussing femininity, masculinity and androgyny, measured with the Positive-Negative Sex-Role Inventory (PN-SRI) [5, 6] within the Gothenburg H70 Birth Cohort Study (the H70 study). Since the launching of the term ‘gender expression’ [1], studies are able to distinguish between the interrelated terms gender expression (femininity, masculinity, androgyny) and gender roles. This disentanglement is advantageous as gender roles comprise a wider concept of socially constructed roles based on societal gender-related norms (e.g. family obligations, work-family conflict or life style activities) [4]. Giving attention to both sex and gender perspectives is critical for valid scientific research on population health, especially within psychopathology research [7]. Although sex and gender entities (e.g. gender expression, gender roles) are connected, they need to be differentiated in order to improve our understanding of disease etiology, which can inform the development of appropriate assessments [7] and strategies for prevention and treatment [8].

Depression is a common disorder that is about twice as common in women compared to men throughout life, independent of age, time period, and cultural setting [9]. However, a cross-national study [10] suggested differences in the magnitude of the sex ratio, which may be caused by an internationally unequal distribution of social determinants in health (e.g. education, unemployment), and unequal rights (e.g. women’s autonomy, legal rights regarding sexual orientation). The sex ratio is present across the life course (after puberty) [11]. However, the ratio decreases with age [12], starting with a post-menopausal decline in women [13], and continue to decrease after age 65 [14]. Thus, the sex ratio is still present in late life, but at a lower level [15, 16]. Mechanisms underlying the sex ratio in depression prevalence are debated [10]. Common explanations include low male help-seeking [17] and reporting [18] behavior, methodological artefacts causing underdiagnosis of depression in men (e.g. biased measuring instruments or diagnostic tools) [18–20], biological differences (e.g. hormones or genetics) [21], social factors (e.g. discrimination, or socioeconomic disadvantages) [22] and gender-related factors (e.g. gender roles or gender expression) [23].

Previous studies regarding gender expression in relation to depression are scarce. However, it has been proposed that there is an association between gender expression and depression. Available evidence is primarily generated from studies conducted among students during the late 20^th^ century, and studies to date have estimated depression using symptom scores. Diagnostic assessments in accordance with the Diagnostic and Statistical Manual of Mental Disorders (DSM) criteria [24–26] are lacking. Among adolescents and younger adults, lower levels of depressive symptoms are most often associated with endorsing an androgynous [27–29], or masculine [29–33] gender expression, although one study found an association between both lower levels [33] and higher levels [28, 29] of depressive symptoms in relation to femininity. Few studies have examined the association between depression and gender expression among older adults. Among older populations, endorsing a masculine [34] or an androgynous [35] gender expression is suggested to be associated with lower rates of depression.

Previous studies suggest that by including socially undesirable aspects of gender expression, unique contributions can be generated in the understanding of gender-related differences in health outcomes [5]. In this study, gender expression is operationalized with the Positive-Negative Sex-Role Inventory (PN-SRI) [5, 6], which conceptualizes three gender expressions (femininity, masculinity and androgyny). The PN-SRI contains both socially desirable gender-coded traits (stereotypical masculine traits e.g. practical or solution-focused; stereotypical feminine traits e.g. empathic or sensitive) and, in contrast to most previous inventories, also socially undesirable gender-coded traits (stereotypical masculine traits e.g. arrogant or inconsiderate; stereotypical feminine traits e.g. cautious or self-doubting).

Based on previous findings, we hypothesized that masculinity and androgyny would be associated with lower, while femininity would be associated with higher, prevalence of depression and burden of symptoms. We also hypothesized that undesirable traits would be related to higher prevalence of depression. Thus, we did not expect that findings among older adults would differ from those derived from studies including younger age-groups. Importantly however, this study adds to a scarcely studied association, for which more evidence is needed in order to evaluate whether or not gender expression may be a relevant factor when studying sex differences in late life depression.

We aimed to test if gender expression (femininity, masculinity, and androgyny) is associated with depression and burden of depressive symptoms in a 70-year-old population.

## Methods and materials

### Study sample

**Fig 1:**
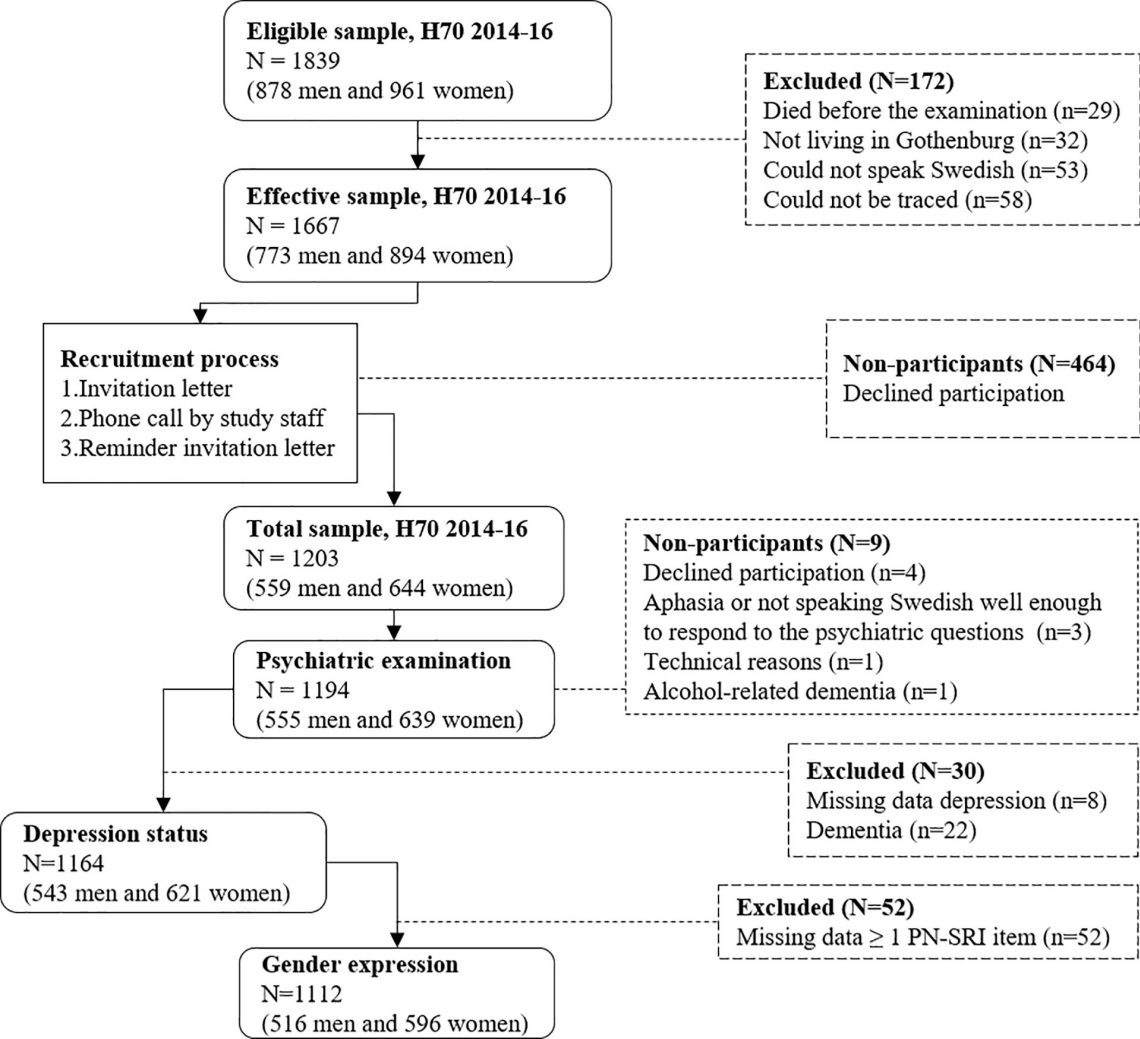
Study sample flow chart. In short, 1203 individuals participated in the study (response rate 72.2%). Out of these, 1194 took part in a psychiatric examination. In this paper, participants for whom depression diagnosis could not be established due to missing data (n = 8) and participants with dementia (n = 22) were excluded, leaving 1164 individuals. Out of these, participants with missing data for ≥ 1 PN-SRI items were excluded from the analyses (n = 52), leaving 1112 individuals.

The study is part of the Gothenburg H70 Birth Cohort Studies in Sweden (the H70 studies), previously described in detail [36]. In 2014–16, all 70-year-olds registered as residents in Gothenburg and born during 1944 on birth dates ending with 0, 2, 5 or 8 were eligible, and 1203 participated (response rate 72.2%). Information regarding date of birth and residential address were obtained from the Swedish Tax Agency’s population register, covering all persons registered as living in Sweden. Persons were considered eligible irrespective of place of residence (e.g. private households, sheltered living). Participant flowchart is displayed in Fig 1.

### Study procedures

The examinations were conducted at an outpatient clinic or in the participant’s home [36], and comprised comprehensive social, somatic, cognitive, functional, and psychiatric examinations, self-rated questionnaires, close informant interviews and a battery of laboratory tests. Examinations were performed by health professionals. The H70 study 2014–16 was approved by the Regional Ethical Review Board (Dnr 869–13). Informed written consent was obtained from all participants or their close relatives, and the study was conducted according to the Helsinki Declaration.

### Psychiatric examination

Psychiatric examinations included ratings of psychiatric symptoms (reported from the participant) and signs (observed by the interviewer) during the past month according to the Comprehensive Psychopathological Rating Scale (CPRS) [37], which has good reliability among older persons [38]. Depressive symptom burden was rated according to the Montgomery-Åsberg Depression Rating Scale (MADRS) [39], which is a subscale of the CPRS including ten depressive symptoms (inner tension, inability to feel, pessimistic thoughts, suicidal thoughts, lassitude, concentration difficulties, reduced appetite, reduced sleep, and reported and observed sadness). Individual items were rated from 0 (no symptoms) to 6 (severe symptoms), generating a score ranging 0–60.

Prior to the study, inter-rater reliability was assessed in 179 individuals who had dual ratings by either psychiatric nurses or psychiatrists. Kappa values for the individual depressive symptoms in MADRS ranged between 0.50 and 0.78 indicating a “moderate agreement” [40] (0.50 (observed sadness); 0.54 (reduced sleep); 0.60 (concentration difficulties)), or a “substantial agreement” [40] (0.64 (inability to feel); 0.66 (lassitude); 0.67 (inner tension); 0.68 (reduced appetite); 0.70 (pessimistic thoughts); 0.72 (reported sadness); 0.78 (suicidal thoughts)).

### Diagnoses

#### Depression

Depression diagnoses were established using computerized symptom algorithms [41] based on clinical assessments of depressive symptoms within the CPRS. Major depression was diagnosed according to the Diagnostic and Statistical Manual of Mental Disorders Fifth Edition (DSM-5) [26], requiring at least 5 out of 9 pre-specified depressive symptoms occurring during the past month, of which at least one had to be depressed mood or diminished interest/pleasure. Minor depression required the presence of 2–4 symptoms according to DSM-IV-TR research criteria [25]. For the purpose of this paper, the term “any depression” was used to denote those fulfilling criteria for either major or minor depression.

#### Dementia

Dementia diagnosis was based on the criteria in the DSM-III-R [24], using information from neuropsychiatric examinations and close informant interviews, as previously described [42]. The diagnosis of dementia was used for exclusion only.

### Gender expression

The Positive-Negative Sex-Role Inventory (PN-SRI) comprises 24 gender-coded self-reported personality traits on a seven-step scale, ranging from one point (never or almost never true) to seven points (always or almost always true). The scale also contains dimensions of social desirability (desirable or undesirable). The classifications of femininity, masculinity, and social desirability are shown in Table 1, as described previously [5].

**Table 1:** The gender-coded personality traits within the Positive-Negative Sex-Role Inventory (PN-SRI).

Masculinity scale		Femininity scale	
**MAS+ items**	**MAS- items**	**FEM+ items**	**FEM- items**
Analytical	Arrogant	Emotional	Anxious
Logical	Boastful	Empathic	Disoriented
Objective	Harsh	Loving	Naïve
Practical	Inconsiderate	Passionate	Overcautious
Rational	Ostentatious	Sensitive	Oversensitive
Solution-focused	Power-hungry	Tender	Self-doubting

Abbreviations: PN-SRI = Positive-Negative Sex-Role Inventory; FEM(+) = Feminine personality traits (desirable); FEM(-) = Feminine personality traits (undesirable); MAS(+) = Masculine personality traits (desirable); MAS(-) = Masculine personality traits (undesirable).

The femininity and masculinity scales comprise 12 items each. Scale scores range between 12 and 84 points, with higher scores reflecting a higher level of femininity/masculinity. Based on the social desirability classification, femininity comprises two sub-scales: socially desirable feminine personality traits (FEM+) including six items (e.g. empathic, sensitive), and undesirable feminine personality traits (FEM-) including six items (e.g. naive, self-doubting). Corresponding subscales for masculinity are desirable masculine personality traits (MAS+) with six items (e.g. logical, solution-focused), and undesirable masculine personality traits (MAS-) with six items (e.g. arrogant, harsh). Items for each subscale are listed in Table 1. Sub-scale ratings range from 6 to 42 points.

In accordance with previous studies [3], an androgyny t score was calculated as the ratio (Student’s t ratio) between masculine and feminine items (*ratio* = masculinity/femininity), reflecting the relative level of masculinity and femininity in a person’s self-descripted gender expression ([t] ≥ 2.09; df = 22; *p*<0.05). An androgyny difference score was calculated as the difference between masculinity score and femininity score (*difference* = masculinity–femininity). For the purpose of this paper, both the t score and difference score are used in order to facilitate comparison with previous studies. A value closer to 0 on either scale indicates an androgynous gender expression where both masculine and feminine personality traits are endorsed, while higher values indicate a gender-typed expression where either masculine or feminine personality traits are dominant. Absolute values are used in all analyses. PN-SRI has acceptable levels of face validity and reliability among participants in the H70 study [6].

### Covariates

Based on previous studies on depression and gender expression, self-reported covariates were tested as potential confounders. Based on our literature search (S1 Table), covariates included: sex (Man; Woman), education (≤Primary; Secondary; Higher), born in Sweden (No; Yes), living in special housing, e.g. retirement home or care facility (No; Yes), living alone (No; Yes), having partner, i.e. being married/having non-cohabiting or cohabiting partner (No; Yes), having a happy relationship with partner (No; Yes), partner receives informal care from the participant (No; Yes), loss of partner during the past five years due to death or divorce (No; Yes), having children (No; Yes), having ≥ one confidant (No; Yes), having contact with healthcare (i.e. with medical doctor or nurse) during the past 12 months (No; Yes), self-rated health, i.e. good, very good or excellent (No; Yes), current smoker (No; Yes), currently working (No; Yes), being primary breadwinner of household (No; Yes), and financial situation (Difficult to make ends meet; Somewhat easy to make ends meet; Very easy to make ends meet). Overall burden of physical and mental illness was assessed using the Cumulative Illness Rating Scale for Geriatrics (CIRS-G) [43], where medical problems for 14 organ systems were rated by research nurses from 0 (no problems) to 4 (extremely severe problems), ranging between 0 to 56. As the rating of psychiatric illness primarily was based on ratings of depression in our study, the psychiatric illness domain was excluded when assessing CIRS-G as a potential confounder for the relationship between gender expression and depression. Functional independence was assessed using the Barthel Index of Activities of Daily Living (ADL), ranging from 0 to 105 (low function/dependent to high function/ independent).

## Statistical analysis

Pearson’s Chi-square was used to test for differences in proportions. Independent samples t-test was used to test for differences in means. Pearson’s r was used to test the correlation between sex and gender expression. The associations between gender expression and MADRS score and depression (major or minor) were tested using three models. In Model 1, we tested the associations between gender expression and depression without adjusting for any covariates. In Model 2, we tested if the associations would be modified when adding sex as a covariate, as sex and gender expression are theoretically interrelated. In Model 3, we further tested the associations between gender expression and depression by adding the full set of selected covariates. Linear regression was used to test the associations between gender expression and MADRS score (Model 1), with sex as covariate in Model 2. Binary logistic regression was used to test sex differences in the prevalence of depression (crude odds ratios with 95% confidence intervals), and the associations between gender expression and any depression (Model 1), adding sex as covariate in Model 2. To select covariates for the fully adjusted Model 3, linear regression was used to test each potential confounder in relation to MADRS, femininity, masculinity, and androgyny scores. Only covariates that were associated with both depression and gender expression were included.

In order to check for potential effect modification by sex, the interaction terms sex*(femininity, masculinity, androgyny) were added in regressions models.

### Sensitivity analyses

Sensitivity analyses were performed using linear regression models. First, the association between gender expression and MADRS was tested using square root transformed values for MADRS score in order to reduce the effects of non-normal distribution. Second, gender expression was added as covariate when analyzing the association between sex and depression, in order to test if the association would change. Third, the association between gender expression and MADRS was tested after excluding all participants with a depression diagnosis, in order to exclude the influence of depression on the self-rated responses regarding gender expression.

Statistical methods were carried out using the IBM SPSS STATISTICS 22 and R program for data transformation and computation (R version 3.6.0 package dplyr for data wrangling). *P*-values <0.05 (two-tailed) were considered statistically significant.

## Results

### Sex differences in depression and gender expression

Table 2 shows the sample characteristics. Women had a higher MADRS score, total femininity score, femininity score with socially desirable (FEM+ score) and undesirable (FEM- score) traits, and a higher prevalence of any depression, compared to men. Men had a higher total masculinity score, and masculinity score with socially desirable (MAS+ score) and undesirable (MAS- score) traits, compared to women. Correlations between sex and gender expressions ranged between -0.19 to 0.24 (S2 Table). Correlations between MADRS score and the specific PN-SRI items ranged between -0.12 to 0.26 (S3 Table).

**Table 2:** Sample characteristics of 70-year-olds (born 1944, examined in 2014–16) by sex.

	Women	Men	All	Sex differences		
% (no. of cases/ total sample)	53.4 (621/1164)	46.6 (543/1164)	100.0 (1164)			
**Demographics**						**p**^a^
Born in Sweden	**87.3** (541/620)	**83.1** (451/543)	**85.3** (992/1163)	---	---	***
Living alone	**43.6** (271/621)	**27.8** (151/543)	**36.3** (422/1164)	---	---	***
Special housing	**0.6** (4/621)	**1.1** (6/543)	**0.9** (10/1164)	---	---	0.40
**Education**						
Primary education	**11.9** (74/621)	**16.8** (91/543)	**14.2** (165/1164)	---	---	***
Secondary education	**49.3** (306/621)	**40.7** (221/543)	**45.3** (527/1164)	---	---	***
Higher education	**38.8** (241/621)	**42.5** (231/543)	**40.5** (472/1164)	---	---	0.21
**Work status**						
Working now	**12.6** (78/621)	**23.2** (126/543)	**17.5** (204/1164)	---	---	***
**Financial situation**						
Primary breadwinner	**54.9** (241/439)	**83.9** (338/403)	**68.8** (579/842)	---	---	***
Difficult to make ends meet	**17.7** (105/593)	**10.1** (52/513)	**14.2** (157/1106)	---	---	***
Somewhat easy to make ends meet	**45.7** (271/593)	**43.9** (225/513)	**44.8** (496/1106)	---	---	0.54
Very easy to make ends meet	**36.6** (217/593)	**46.0** (236/513)	**41.0** (453/1106)	---	---	***
**Relationships**						
Having partner	**64.1** (398/621)	**83.8** (455/543)	**73.3** (853/1164)	---	---	***
Happy relationship	**51.5** (205/398)	**53.4** (243/455)	**52.5** (448/853)	---	---	***
Personal care of partner	**5.0** (31/619)	**2.4** (13/543)	**5.2** (44/853)	---	---	***
Lost partner preceding 5 y	**5.2** (32/621)	**4.1** (22/543)	**4.6** (54/1164)	---	---	0.37
Having children	**87.0** (535/615)	**87.4** (470/538)	**87.2**(1005/1153)	---	---	0.85
Having ≥ 1 confidant	**95.3** (573/601)	**88.1** (459/521)	**92.0**(1032/1122)	---	---	***
**Health**				**t** ^(mean diff.)^	**(95% CI)**	**p**^c^
Healthcare in preceding y	**93.4** (566/606)	**92.7** (496/535)	**93.1**(1062/1141)	---	---	0.65
Good/Excellent self-rated health	**82.1** (494/602)	**86.5** (449/519)	**84.1** (943/1121)	---	---	***
Smoking now	**10.9** (67/617)	**7.2** (39/542)	**9.1** (106/1159)	---	---	***
CIRS-G score, mean ^(SD)^	6.1 ^(4.1)^	5.9 ^(3.9)^	5.9 ^(4.0)^	-0.7 ^(-0.2)^	-0.65–0.30	0.47
ADL score, mean ^(SD)^	103.1 ^(6.9)^	103.6 ^(5.8)^	103.3 ^(6.4)^	1.4 ^(0.5)^	-0.20–1.29	0.15
**Depression**				**OR**	**(95% CI)**	**p**^b^
Major depression	**3.2** (20/621)	**1.5** (8/543)	**2.4** (28/1164)	2.2	0.97–5.09	0.06
Minor depression	**7.6** (47/621)	**5.2** (28/543)	**6.4** (75/1164)	1.5	0.93–2.44	0.10
Any depression	**10.8** (67/621)	**6.6** (36/543)	**8.8** (103/1164)	1.7	1.12–2.60	***
**Burden of depressive symptoms**				**t** ^(mean diff.)^	**(95% CI)**	**p**^c^
MADRS score, mean ^(SD)^	4.4 ^(5.4)^	3.6 ^(4.6)^	4.0 ^(5.1)^	2.8 ^(0.8)^	0.23–1.39	***
**%** (no. of cases/ total cases)	**53.6** (596/1112)	**46.4** (516/1112)	**100.0** (1112)			
**Femininity**				**t** ^(mean diff.)^	**(95% CI)**	**p**^c^
Femininity score, mean ^(SD)^	49.5 ^(9.0)^	45.3 ^(8.6)^	47.5 ^(9.0)^	7.8 ^(4.1)^	3.13–5.20	***
FEM+ score, mean ^(SD)^	31.0 ^(5.1)^	28.4 ^(5.5)^	29.8 ^(5.5)^	8.3 ^(2.7)^	2.02–3.28	***
FEM- score, mean ^(SD)^	18.4 ^(6.4)^	16.9 ^(5.8)^	17.7 ^(6.2)^	4.1 ^(1.5)^	0.78–2.21	***
**Masculinity**				**t** ^(mean diff.)^	**(95% CI)**	**p**^c^
Masculinity score, mean ^(SD)^	42.7 ^(7.4)^	45.5 ^(8.2)^	44.0 ^(7.9)^	-5.9 ^(-2.7)^	-3.67–-1.83	***
MAS+ score, mean ^(SD)^	30.6 ^(5.2)^	31.7 ^(5.6)^	31.1 ^(5.4)^	-3.25 ^(-1.05)^	-1.69–-0.42	***
MAS- score, mean ^(SD)^	12.1 ^(5.0)^	13.8 ^(5.2)^	12.9 ^(5.2)^	-5.55 ^(-1.70)^	-2.30–-1.10	***
**Androgyny**				**t** ^(mean diff.)^	**(95% CI)**	**p**^c^
Androgyny t score, mean ^(SD)^	1.2 ^(1.0)^	1.0 ^(0.9)^	1.1 ^(1.0)^	3.1 (0.17)	0.06–0.28	***
Androgyny diff score, mean ^(SD)^	10.0 ^(7.6)^	8.2 ^(6.2)^	9.1 ^(7.0)^	4.4 (1.81)	0.99–2.61	***

Abbreviations: MADRS = Montgomery Åsberg Depression Rating Scale; FEM(+) = Feminine personality traits (desirable); FEM(-) = Feminine personality traits (undesirable); MAS(+) = Masculine personality traits (desirable); MAS(-) = Masculine personality traits (undesirable); Androgyny t score = t statistic ratio of masculinity vs. femininity; Androgyny diff score = difference between masculinity score and femininity score; Androgynous = masculinity vs. femininity (*p*<0.05); SD = standard deviation; OR = odds ratio

*** <0.05.

^a^ Pearson’s chi-square

^b^ Logistic regression (women vs. men)

^c^ Independent samples t-test (women vs. men).

### Gender expression and depression

Table 3(A) shows the association between gender expression and depression. In Model 1, total femininity score and FEM- were positively, while MAS+ was negatively associated with depression. The strengths of associations did not change when adjusting for sex in Model 2. In the fully adjusted model (Model 3), total femininity score, FEM+, and FEM- were positively associated with depression. Table 3(B) shows the association between gender expression and MADRS score. In Model 1, MADRS score was higher among those with feminine personality traits (especially low FEM-), and those with lower androgyny scores, while MADRS score was lower among those with higher MAS+. All associations remained in Model 2 and 3. In addition, positive associations appeared between MADRS score and MAS- in Model 2, and with FEM+ in Model 3.

**Table 3:** Associations between gender expression and a) depression status and b) MADRS score in 70-year-old men and women.

	Model 1					Model 2					Model 3				
**(a) Any depression**^**1**^															
	**OR**	**B**	**SE**	**p**	**95% CI**	**OR**	**B**	**SE**	**p**	**95% CI**	**OR**	**B**	**SE**	**p**	**95% CI**
**Femininity**															
Femininity score	1.05	0.05	0.01	***	1.02–1.07	1.04	0.04	0.01	***	1.02–1.07	1.03^a^	0.03	0.01	***	1.01–1.06
FEM+ score	1.04	0.04	0.02	0.05	0.99–1.08	1.03	0.03	0.02	0.17	0.99–1.07	1.04^b^	0.04	0.02	***	1.01–1.09
FEM- score	1.07	0.07	0.02	***	1.04–1.11	1.07	0.07	0.02	***	1.03–1.10	1.04^c^	0.04	0.02	***	1.003–1.08
**Masculinity**															
Masculinity score	0.98	-0.12	0.01	0.19	0.96–1.01	0.99	-0.01	0.01	0.37	0.96–1.02	0.99^d^	-0.003	0.02	0.86	0.97–1.03
MAS+ score	0.96	-0.04	0.02	***	0.92–0.99	0.96	-0.04	0.02	0.06	0.93–1.00	0.99^e^	-0.01	0.02	0.51	0.95–1.03
MAS- score	1.00	0.01	0.02	0.84	0.96–1.05	1.01	0.01	0.02	0.53	0.97–1.06	1.02^f^	0.02	0.02	0.47	0.97–1.06
**Androgyny**															
Androgyny t score	1.21	0.19	0.10	0.07	0.99–1.47	1.18	0.16	0.10	0.11	0.96–1.44	1.01^g^	0.01	0.11	0.94	0.81–1.26
Androgyny diff score	1.03	0.03	0.01	0.07	0.99–1.06	1.02	0.02	0.01	0.14	0.99–1.05	1.00^g^	0.001	0.02	0.93	0.97–1.03
**(b) MADRS score**^**2**^															
	**R**^**2**^	**B**	**SE**	**p**	**95% CI**	**R**^**2**^	**B**	**SE**	**p**	**95% CI**	**R**^**2**^	**B**	**SE**	**p**	**95% CI**
**Femininity**															
Femininity score	0.042	0.11	0.02	***	0.08–0.14	0.041	0.11	0.02	***	0.08–0.14	0.140^a^	0.09	0.02	***	0.05–0.12
FEM+ score	0.002	0.04	0.03	0.18	-0.02–0.09	0.005	0.02	0.03	0.46	-0.03–0.08	0.076^b^	0.05	0.03	0.09	-0.01–0.11
FEM- score	0.069	0.21	0.02	***	0.17–0.26	0.070	0.21	0.02	***	0.16–0.25	0.162^c^	0.16	0.02	***	0.11–0.20
**Masculinity**															
Masculinity score	0.003	-0.04	0.02	0.05	-0.07–0.00	0.007	-0.03	0.02	0.13	-0.07–0.01	0.114^d^	-0.01	0.02	0.71	-0.05–0.03
MAS+ score	0.018	-0.12	0.03	***	-0.18–-0.07	0.021	-0.12	0.03	***	-0.17–-0.06	0.128^e^	-0.06	0.03	***	-0.11–-0.01
MAS- score	0.003	0.05	0.03	0.09	-0.01–0.11	0.009	0.06	0.03	***	0.01–0.12	0.047^f^	0.07	0.03	***	0.02–0.13
**Androgyny**															
Androgyny t score	0.020	0.73	0.15	***	0.43–1.03	0.022	0.70	0.16	***	0.39–1.00	0.116^g^	0.42	0.15	***	0.13–0.72
Androgyny diff score	0.013	0.08	0.02	***	0.04–0.12	0.015	0.07	0.02	***	0.03–0.12	0.113^g^	0.04	0.02	***	0.01–0.08

^1^ Logistic regression.

^2^ Linear regression. Abbreviations: MADRS = Montgomery Åsberg Depression Rating Scale; FEM(+) = Feminine personality traits (desirable); FEM(-) = Feminine personality traits (undesirable); MAS(+) = Masculine personality traits (desirable); MAS(-) = Masculine personality traits (undesirable); Androgyny t score = t statistic ratios of masculinity vs. femininity; Androgyny diff score = difference between masculinity score and femininity score; OR = odds ratio; SE = Standard Error

*** <0.05. **Model 1** (unadjusted); **Model 2** (adjusted for sex only); **Model 3** (fully adjusted).

^a^Adjusted for sex, living alone, having partner, partner loss, financial situation (making ends meet), and self-rated health.

^b^Adjusted for sex, living alone, happy marriage, financial situation (making ends meet), and having confidant.

^c^Adjusted for sex, living alone, having partner, happy marriage, having children, financial situation (making ends meet), and self-rated health.

^d^Adjusted for sex, living alone, having partner, financial situation (making ends meet), and self-rated health.

^e^Adjusted for sex, living alone, having partner, happy marriage, financial situation (making ends meet), and self-rated health.

^f^Adjusted for sex and financial situation (making ends meet).

^g^Adjusted for sex, financial situation (making ends meet), and self-rated health.

There were no interactions between sex and gender expression in relation to depression or MADRS score (S4 Table), indicating that effects of gender expression were similar in men and women.

The main results for stratifications by sex are shown in Table 4A, 4B. The complete table is presented in S5 Table. In Model 1 (Table 4A), total femininity score and FEM- were positively associated with depression in both women and men. When adding covariates in Model 3, the strengths of associations did not change, but the p-values remained significant among men only. Table 4B shows that, in both sexes, MADRS score was higher among those with higher total femininity score, FEM- score, and androgyny t score, while it was lower among those with higher MAS+ score (Model 1). In Model 3, the associations remained for total femininity score and FEM-, but not for MAS+. The association with androgyny remained in women only.

**Table 4:** Associations between gender expression and a) depression status and b) MADRS score by sex.

	(a) Any depression^1^										(b) MADRS score^2^									
	Model 1					Model 3					Model 1					Model 3				
**Women**																				
	**OR**	**B**	**SE**	**p**	**95% CI**	**OR**	**B**	**SE**	**p**	**95% CI**	**R**^**2**^	**B**	**SE**	**p**	**95% CI**	**R**^**2**^	**B**	**SE**	**p**	**95% CI**
**Femininity**																				
Femininity score	1.04	0.04	0.02	***	1.01–1.07	1.02^a^	0.02	0.02	0.15	0.99–1.06	0.033	0.11	0.02	***	0.06–0.15	0.107^a^	0.08	0.02	***	0.03–0.13
FEM+ score	1.03	0.03	0.03	0.25	0.98–1.09	1.05^b^	0.04	0.03	0.15	0.99–1.10	0.001	0.03	0.04	0.43	-0.05–0.12	0.06^b^	0.06	0.04	0.16	-0.02–0.14
FEM- score	1.05	0.05	0.02	***	1.01–1.10	1.03^c^	0.03	0.02	0.26	0.98–1.07	0.05	0.19	0.03	***	0.12–0.25	0.116^c^	0.14	0.03	***	0.08–0.20
**Men**																				
	**OR**	**B**	**SE**	**p**	**95% CI**	**OR**	**B**	**SE**	**p**	**95% CI**	**R**^**2**^	**B**	**SE**	**p**	**95% CI**	**R**^**2**^	**B**	**SE**	**p**	**95% CI**
**Femininity**																				
Femininity score	1.10	0.05	0.02	***	1.01–1.10	1.05 ^a^	0.05	0.02	***	1.01–1.10	0.043	0.11	0.02	***	0.06–0.15	0.185^a^	0.10	0.02	***	0.06–0.14
FEM+ score	1.03	0.03	0.03	0.44	0.96–1.10	1.05 ^b^	0.05	0.04	0.18	0.98–1.13	0.000	0.01	0.04	0.82	-0.06–0.08	0.088^b^	0.03	0.04	0.39	-0.04–0.10
FEM- score	1.10	0.09	0.03	***	1.03–1.17	1.09 ^c^	0.08	0.04	***	1.02–1.17	0.09	0.24	0.03	***	0.17–0.30	0.223^c^	0.20	0.03	***	0.13–0.26

^1^ Logistic regression.

^2^ Linear regression. Abbreviations: MADRS = Montgomery Åsberg Depression Rating Scale; FEM(+) = Feminine personality traits (desirable); FEM(-) = Feminine personality traits (undesirable); MAS(+) = Masculine personality traits (desirable); MAS(-) = Masculine personality traits (undesirable); Androgyny t score = t statistic ratios of masculinity vs. femininity; Androgyny diff score = difference between masculinity score and femininity score; OR = odds ratio; SE = Standard Error

*** <0.05. **Model 1** (unadjusted); **Model 3** (fully adjusted).

^a^Adjusted for sex, living alone, having partner, partner loss, financial situation (making ends meet), and self-rated health.

^b^Adjusted for sex, living alone, happy marriage, financial situation (making ends meet), and having confidant.

^c^Adjusted for sex, living alone, having partner, happy marriage, having children, financial situation (making ends meet), and self-rated health.

^d^Adjusted for sex, living alone, having partner, financial situation (making ends meet), and self-rated health.

^e^Adjusted for sex, living alone, having partner, happy marriage, financial situation (making ends meet), and self-rated health.

^f^ Adjusted for financial situation (making ends meet).

^g^Adjusted for sex, financial situation (making ends meet), and self-rated health.

**Fig 2:**
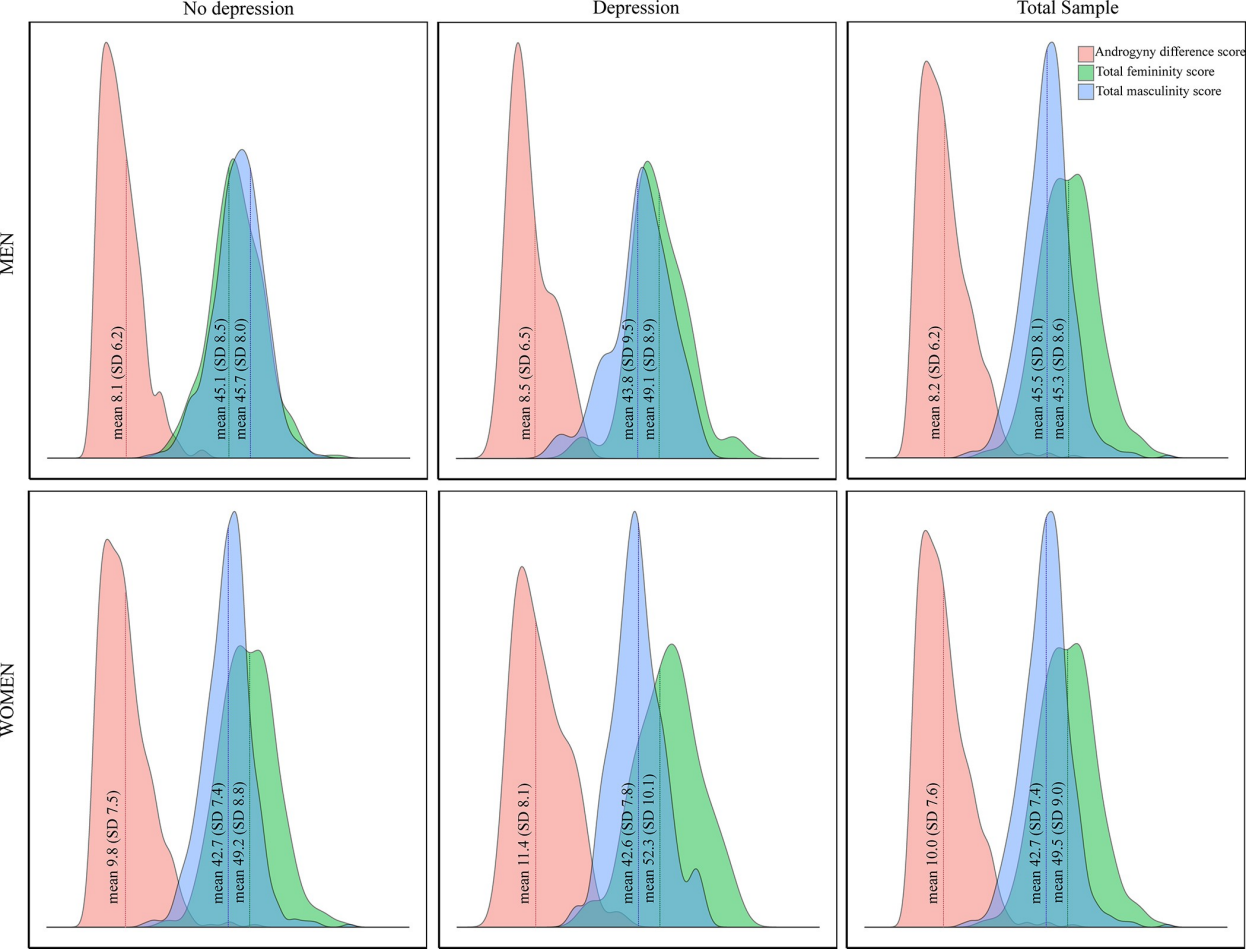
Mean femininity, masculinity and androgyny scores by depression status and sex. Differences in mean values were tested using independent samples t-tests. (1) Within-sex-comparison: Men with depression vs. no depression (androgyny *p* = 0.77; femininity *p*<0.05; masculinity *p* = 0.21). Women with depression vs. no depression (androgyny *p* = 0.12; femininity *p*<0.05; masculinity *p* = 0.88). (2) Between-sex-comparison: Men vs. women without depression (androgyny *p*<0.05; femininity *p*<0.05; masculinity *p*<0.05). Men vs. women with (androgyny *p* = 0.09; femininity *p* = 0.15; masculinity *p* = 0.52).

Mean levels of total femininity score, total masculinity score and androgyny difference score by depression status and sex are shown in Fig 2. The mean femininity score was higher among those with depression compared to those without depression, both among men (49.1 vs. 45.1) and among women (52.3 vs. 49.2). Among those without depression, women had higher mean femininity score (49.2 vs. 45.1) and androgyny difference score (9.8 vs. 8.1) compared to men, while men had higher masculinity score (45.7 vs. 42.7) compared to women. There were no sex differences in gender expression among those with depression. Within-sex differences were observed for neither masculinity nor androgyny.

### Sensitivity analyses

First, the association between gender expression and square root transformed values for MADRS score supported the results shown in Models 1–3. Second, when adding FEM- as a covariate, the association between sex and depression changed; the effect sizes decreased and the p-values increased (Any depression: OR 1.75; *p*<0.05 to OR 1.57; *p* = 0.05 and MADRS score: B 0.78; *p*<0.05 to B 0.47; p = 0.11). The other gender expressions did not influence the association between sex and depression (S4 Table). Third, excluding participants with depression supported the overall results shown in Models 1–3, although some associations between gender expression and MADRS score were modified (S1 Text).

## Discussion

In this cross-sectional population study of 70-year-olds, we found that gender expression was related to depression and burden of depressive symptoms. In line with our hypotheses, femininity (especially traits with low social desirability) were related to a higher prevalence of depression and a higher level of depressive symptoms, in both women and men. In addition, when testing the role of FEM- in the association between sex and depression, the effect of biological sex decreased. Further in line with our hypothesis, we found that androgyny and masculine traits with high social desirability were associated with lower burden of depressive symptoms. However, we failed to show the hypothesized association with depression (major or minor).

Our findings highlight the importance of taking both sex and gender expression into consideration when studying the epidemiology of depression. Previous studies testing the association between gender expression and depression are scarce. Most studies have primarily used the Bem Sex Role Inventory (BSRI) [3] to measure gender expression, which only includes socially desirable gender expression traits. Thus, comparisons with the results of our study are difficult, as we are the first to employ the PN-SRI in relation to depression among older adults. Further, only two studies have evaluated the relationship between gender expression and depression in older adults [34, 35]. A cross-national study (age 65–74) from Canada, Albania, Colombia and Brazil (conducted in 2012) reported that androgyny was associated with lower rates of depressive symptoms, when adjusting for biological sex [35]. We found that a more androgynous gender expression was related to lower burden of depressive symptoms which parallels to the androgyny model. The androgyny model proposes that androgynous persons are more flexible in their behavioral and psychological adaptability, compared to masculine males and feminine females (sex-typed individuals) who are more restricted by gender-related norms [44]. This adaptive flexibility has been suggested to be beneficial in terms of mental health [44, 45]. Our findings that higher levels of masculinity were associated with lower depression symptom burden in the total sample expands on previous findings of a Canadian study that focused on women living in nursing homes (age 68–97), conducted during the 1980s [34]. However, in contrast to our study, they found no association between depression and femininity, which may have been due to the small number of participants (n = 30).

Apart from the two studies mentioned above, previous studies assessing the relationship between gender expression and depression have primarily been conducted among Canadian or American students between 1990 and 2005. Also, most studies used other assessments of depression compared to our study (mainly different scales). Despite having a sample of older participants and other assessment instruments for both gender expression and depression, our findings reveal patterns not unlike those demonstrated for young cohorts; femininity was associated with higher levels of depression [28, 29], while masculinity [28–31, 33], and androgyny was related to lower levels of depression [27–29].

Only one longitudinal study has evaluated the association between gender expression and depression, and the study sample was young (age 12–25) [32]. The results showed that increased masculine gender expression trajectories during adolescence and early adulthood were related to lower levels of depressive symptoms at follow-up, in both sexes. However, it is unclear if this finding, which is related to a very specific period in early life, may be relevant for older adults.

Our associations between gender expression and depression were mainly attributable to socially undesirable feminine traits (FEM-), pointing towards the need to include both desirable and undesirable traits when studying various health outcomes in relation to gender expression. Thus, different dimensions of gender expression may have diverse effects on outcomes of depression. When testing the correlation between the PN-SRI feminine traits with the individual depressive symptoms in MADRS, the correlation coefficients were lower than we expected, considering their potential overlaps. The association between depression and gender expression is complex and may display different patterns for men and women. However, we found no interaction between sex and gender expression, suggesting that the association between gender expression and depression was similar in men and women. Interestingly, the association between sex and depression was no longer statistically significant when FEM- was added in the model. These results suggest that feminine traits may be an important factor to consider regarding sex differences in depression etiology and symptom expression. Furthermore, we found that men with higher level of FEM- score had higher odds of having depression compared to other men. This finding points to the importance of considering different aspects of gender expression also within each sex, including both socially desirable and undesirable traits.

Adding to the possibility that femininity is a potential risk factor for depression, our results may imply that those endorsing a feminine gender expression, irrespective of biological sex, may be more prone to verbally express depressive symptoms. Communicating about low mood with friends or family may generate increased emotional support, reducing the risk for depression [46, 47]. Emotional support may, in turn, prevent feeling lonely which is a risk factor for depression [48]. On the contrary, being able to verbalize more about one’s wellbeing may also partly explain the association between femininity and the higher level of depression in this study, as this may generate a higher likelihood of being detected to have depression. In addition, studies have suggested that masculinity-related norms become barriers for disclosing low mood, in order to avoid ‘looking weak’ [18, 49, 50]. Also, older persons have disclosed experiencing a reduced ability to solve problems in daily life while having depression [51]. As ‘problem-solving’ is a masculine trait with high social desirability in the PN-SRI, the association between MAS+ and lower level of depression may be partly attributed to having problem-solving skills. However, these hypotheses need to be further studied before any conclusions can be drawn.

When including measures of gender expression in research, results must be interpreted with caution as norms and attitudes regarding gender expression can vary across cultures and over time. Further, reducing gender expression to a fixed set of attributes, as in PN-SRI, requires serious consideration of differences and similarities that are structured by the instrument. Also, there is a risk of reinforcing gender stereotypes when put in focus. In epidemiology, however, it is crucial to clearly define and classify all studied phenomena in order to facilitate the collection of data and avoid biased interpretations [52]. In addition, with or without a gender expression measurement, gender norms and preconceptions about stereotypical male and female attributes will still be present in our everyday life, affecting our health. With this in mind, the pros of studying gender expression may outweigh the cons, as including gender expression can give a unique contribution of knowledge when studying sex differences in health.

As depression is heterogenous regarding both etiology and symptom expression, it is not only important to reveal potential differences between men and women, but also the potential differences within each sex. Those endorsing a masculine gender expression, irrespective of biological sex, may be less prone to be diagnosed with depression. Measurement and diagnostic instruments have been suggested to suffer from a ‘catch 22’; capturing a female phenotype of depression [18], and thereby underestimating depression among those expressing male phenotype symptoms, as these symptoms are not included in the diagnostic criteria [53]. A male phenotype may include externalizing symptoms (e.g. substance use, risk-taking, and aggression), and may reflect behavioral manifestations of masculine norms in relation to depression [19]. Thus, having a gender perspective in the health care of depressed patients could nuance preconceptions about sex differences in the occurrence, etiology and symptom expression of depression, and increase the detection of depression in patients with atypical symptom patterns. Future research should use a longitudinal design to validate temporality. The independent roles of sex and gender expression in relation to depression should be studied in various societal settings due to differences in norms and attitudes across cultural contexts. Furthermore, studies on how gender expression traits may affect not only the susceptibility to depression during older age, but also depression-related impairment and treatment response, are warranted.

### Strengths and limitations

Strengths of this study include the comprehensive personal examinations, the representative population-based sample, and the high response rate, which reduces the risk of sampling bias compared to samples derived from e.g. clinical settings. Depression diagnoses were based on psychiatric examinations conducted by psychiatrists, medical doctors or psychiatric nurses. In addition, our diagnoses are based on past month symptoms, which reduces recall bias. Also, utilizing PN-SRI as a measure of gender expression enabled the study of not only socially desirable but also undesirable gender coded traits.

There are also some limitations. First, the study is cross-sectional. The direction of the association between gender expression and depression can thus not be clearly elucidated. Second, no distinction was made between major and minor depression. Some sub-groups were small, limiting the statistical power which may have generated false negative results. Also, there is a risk of Type 1 error as we did not perform a Bonferroni correction. Third, by utilizing the PN-SRI, gender expression is reduced to a fixed set of 24 self-reported personality traits, which may not fully capture the complexity of the phenomenon. Fourth, due to stigma as well as political awareness, the gender coded personality traits are potentially at risk for social desirability bias. It is not clear how social desirability bias might have affected the associations with depression.

## Conclusions

We found that gender expression was associated to both depression and burden of depressive symptoms. More specifically, we found that femininity was associated to higher levels of depression, irrespective of biological sex. In addition, masculinity and androgyny were associated with lower levels of depression. These results highlight the importance of taking gender expression into consideration when studying sex differences in depression among older populations in future studies.

## Supporting information

S1 Table Protocol for literature search and selection of potential covariates. (DOCX)

S2 Table Correlations between sex and gender expression. Correlation (Pearson). Abbreviations: FEM(+) = Feminine personality traits (desirable); FEM(-) = Feminine personality traits (undesirable); MAS(+) = Masculine personality traits (desirable); MAS(-) = Masculine personality traits (undesirable); Androgyny t score = t statistic ratios of masculinity vs. femininity; Androgyny diff score = difference between masculinity score and femininity score; *** <0.05. ^a^ n = 1112. ^b^ n = 91. ^c^ n = 1021.
(DOCX)

S3 Table Correlations between MADRS and gender expression. Correlation (Pearson). Abbreviations: FEM(+) = Feminine personality traits (desirable); FEM(-) = Feminine personality traits (undesirable); MAS(+) = Masculine personality traits (desirable); MAS(-) = Masculine personality traits (undesirable).
(DOCX)

S4 Table Association between sex and a) depression status and b) MADRS score (step ^1^), adding gender expression as covariate (step ^2^) and as potential effect modifier (step ^3^). ^1^ Logistic regression. ^2^ Linear regression. **Step** ^**1**^ = Association between sex and depression (unadjusted model); **Step** ^**2**^ = Association between sex and depression, adding gender expression as covariate; **Step** ^**3**^ = Gender expression was tested as a potential effect modifier on the association between sex, any depression, and MADRS score (sex*gender expression). Abbreviations: MADRS score = burden of depressive symptoms; FEM(+) = Feminine personality traits (desirable); FEM(-) = Feminine personality traits (undesirable); MAS(+) = Masculine personality traits (desirable); MAS(-) = Masculine personality traits (undesirable); Androgyny t score = t statistic ratios of masculinity vs. femininity; Androgyny diff score = difference between masculinity score and femininity score; SE = Standard Error; *** <0.05.
(DOCX)

S5 Table Associations between gender expression and a) depression status and b) MADRS score by sex (complete Table 4). ^1^ Logistic regression. ^2^ Linear regression. Abbreviations: MADRS = Montgomery Åsberg Depression Rating Scale; FEM(+) = Feminine personality traits (desirable); FEM(-) = Feminine personality traits (undesirable); MAS(+) = Masculine personality traits (desirable); MAS(-) = Masculine personality traits (undesirable); Androgyny t score = t statistic ratios of masculinity vs. femininity; Androgyny diff score = difference between masculinity score and femininity score; OR = odds ratio; SE = Standard Error; *** <0.05. Model 1 (unadjusted); Model 3 (fully adjusted). ^a^Adjusted for sex, living alone, having partner, partner loss, financial situation (making ends meet), and self-rated health. ^b^Adjusted for sex, living alone, happy marriage, financial situation (making ends meet), and having confidant. ^c^Adjusted for sex, living alone, having partner, happy marriage, having children, financial situation (making ends meet), and self-rated health. ^d^Adjusted for sex, living alone, having partner, financial situation (making ends meet), and self-rated health. ^e^Adjusted for sex, living alone, having partner, happy marriage, financial situation (making ends meet), and self-rated health. ^f^ Adjusted for financial situation (making ends meet). ^g^Adjusted for sex, financial situation (making ends meet), and self-rated health.
(DOCX)

S1 Text Third sensitivity analysis. (DOCX)
