# Supplementary material for: Depression in relation to sex and gender expression among Swedish septuagenarians—Results from the H70 study
Source: PLoS One. 2020 Sep 14;15(9):e0238701. doi: 10.1371/journal.pone.0238701 (PMC7489509; doi:10.1371/journal.pone.0238701)
Supplement: S1 Table — (DOCX) [file pone.0238701.s001.docx]

**Protocol – (1) literature search and (2) selection of potential confounders**

**(1) Literature search**

*Older adults*

- **Search in Scopus database (2019-12-23)**: (TITLE (Depress*)) AND (TITLE-ABS-KEY (elder* OR “older persons” OR “older adults” OR “aging” OR “ageing” OR “gerontology”) AND (“gender”) AND (“femininity” OR “masculinity” OR “androgyny”)).
- **Audit**: Abstract and/or full article audit.
- **Inclusion criteria**: quantitative design, older adults (>65 years), dependent/independent variables depression and femininity, masculinity, androgyny.
- **Result**: Out of 65, 2 publications met our inclusion criteria.

1. Vafaei A, Ahmed T, Freire Ado N, Zunzunegui MV, Guerra RO. Depression, Sex and Gender Roles in Older Adult Populations: The International Mobility in Aging Study (IMIAS). PLoS One 2016; 11(1): e0146867.
2. Krames L, England R, Flett GL. The Role of Masculinity and Femininity in Depression and Social Satisfaction in Elderly Females. Sex Roles 1988; 19(11–12): 713–721.

*All ages*

- **Search in Scopus database (2019-12-23)**: (TITLE (Depress*)) AND (“femininity” OR “masculinity” OR “androgyny”)).
- **Audit**: Abstract and/or full article audit.
- **Inclusion criteria**: quantitative design, dependent/independent variables depression and femininity, masculinity, androgyny.
- **Result**: Out of 1041, 12 publications met our inclusion criteria.

1. Gibson PA, Baker EH, Milner AN. The role of sex, gender, and education on depressive symptoms among young adults in the United States. J Affect Disord 2016; 189: 306–313.
2. Grimmell D. Effects of gender-role self-discrepancy on depressed mood. Sex Roles 1998; 39(3/4).
3. Hunt MG. Expressiveness Does Predict Well-Being. Sex Roles 1993; 29(3–4): 147–169.
4. Krames L, England R, Flett GL. The Role of Masculinity and Femininity in Depression and Social Satisfaction in Elderly Females. Sex Roles 1988; 19(11–12): 713–721.
5. Marcotte D, Alain M, Gosselin MJ. Gender differences in adolescent depression: Gender-typed characteristics or problem-solving skills deficits? Sex Roles 1999; 41(1–2): 31–48.
6. Marcotte D, Fortin L, Potvin P, Papillon M. Gender differences in depressive symptoms during adolescence: Role of gender-typed characteristics, self-esteem, body image, stressful life events, and pubertal status. J Emot Behav Disord 2002; 10(1): 29–42.
7. Pidano AE, Tennen H. Transient depressive experiences and their relationship to gender and sex-role orientation. Sex Roles 1985; 12(1/2).
8. Price EC, Gregg JJ, Smith MD, Fiske A. Masculine traits and depressive symptoms in older and younger men and women. Am J Men’s Health 2018; 12(1): 19-29.
9. Sanfilipo MP. Depression, gender, gender role traits, and the wish to be held. Sex Roles 1993; 28(9/10).
10. Sanfilipo MP. Masculinity, Femininity, and Subjective Experiences of Depression. J Clin Psychol 1994; 50(2): 144–157.
11. Vafaei A, Ahmed T, Freire Ado N, Zunzunegui MV, Guerra RO. Depression, Sex and Gender Roles in Older Adult Populations: The International Mobility in Aging Study (IMIAS). PLoS One 2016; 11(1): e0146867.
12. Lo IPY, Kim YK, Small E, Chan CHY. The Gendered Self of Chinese Lesbians: Self-Esteem as a Mediator Between Gender Roles and Depression. Arch Sex Behav 2019; 48(5): 1543–1554.

**(2) Selection of potential confounders**

In order to include potential confounders, the above publications were audited. The list of covariates included in previous studies is seen in Supplementary Table S1. To select covariates for the fully adjusted (Model 3), linear regression was used to test each potential confounder in relation to depression (MADRS), and gender expression (femininity, masculinity, androgyny scores). Only covariates that were associated with both depression and gender expression were included in Model 3.

**Supplementary Table S1**. List of self-reported covariates tested as potential confounders for the association between gender expression and depression

| **Covariates** | **Coding** | **Association to dependent (depression) and independent (gender expression) variables** |
| --- | --- | --- |
| Sex (*the biological distinction between men and women based on the information given by their Swedish personal identity number*) | Man/Woman | Yes |
| Primary education | Yes/No | No |
| Secondary education | Yes/No | No |
| Higher education | Yes/No | No |
| Born in Sweden | Yes/No | No |
| Living in special housing (*type of residence including e.g. retirement home or care facility*) | Yes/No | No |
| Living alone | Yes/No | Yes |
| Having partner (*i.e. married, or having non-cohabiting or cohabiting partner*) | Yes/No | Yes |
| Happy relationship | Yes/No | Yes |
| Personal care of partner (*i.e. if the partner received informal care from the participant*) | Yes/No | No |
| Lost their partner during the past five years due to death or divorce | Yes/No | Yes |
| Having children | Yes/No | Yes |
| Having at least one confidant | Yes/No | Yes |
| Having contact with health care (*with medical doctor or nurse during the past 12 months*) | Yes/No | No |
| Self-rated health (*good, very good or excellent*) | Yes/No | Yes |
| Smoking (*current smoker*) | Yes/No | No |
| Working (*currently working*) | Yes/No | No |
| Primary breadwinner | Yes/No | No |
| Financial situation (*making ends meet*) | With difficulty/ Somewhat easy/ With ease | Yes |
| Burden of physical illness (*CIRS-G score*) | 0-56 | No |
| ADL (*low function/dependent to high function/independent*) | 0-105 | No |
| **Variables found during publication audit, *not collected* in the H70 study**: parents’ education, adolescent family status, adolescent alcohol use, job stress, locus of control, self-efficacy, self-esteem, parent-child geographic proximity | | |
