# Supplementary material for: Depression in relation to sex and gender expression among Swedish septuagenarians—Results from the H70 study
Source: PLoS One. 2020 Sep 14;15(9):e0238701. doi: 10.1371/journal.pone.0238701 (PMC7489509; doi:10.1371/journal.pone.0238701)
Supplement: S2 Table — Correlation (Pearson). Abbreviations: FEM(+) = Feminine personality traits (desirable); FEM(-) = Feminine personality traits (undesirable); MAS(+) = Masculine personality traits (desirable); MAS(-) = Masculine personality traits (undesirable); Androgyny t score = t statistic ratios of masculinity vs. femininity; Androgyny diff score = difference between masculinity score and femininity score; *** <0.05. a n = 1112. b n = 91. c n = 1021. (DOCX) [file pone.0238701.s002.docx]

**S2 Table**

**Table heading: Correlations between sex and gender expression**

|  | **Total sample**^†^ | | **Depression**^‡^ | | **No depression**^§^ | |
| --- | --- | --- | --- | --- | --- | --- |
|  |  |  |  |  |  |  |
|  | **Coefficient** | **p** | **Coefficient** | **p** | **Coefficient** | **p** |
| **Femininity** |  |  |  |  |  |  |
| Total femininity score | 0.23 | *** | 0.15 | 0.15 | 0.23 | *** |
| FEM+ score | 0.24 | *** | 0.22 | *** | 0.24 | *** |
| FEM- score | 0.12 | *** | 0.04 | 0.72 | 0.12 | *** |
| **Masculinity** |  |  |  |  |  |  |
| Total masculinity score | -0.18 | *** | -0.07 | 0.52 | -0.19 | *** |
| MAS+ score | -0.10 | *** | -0.01 | 0.91 | -0.11 | *** |
| MAS- score | -0.17 | *** | -0.09 | 0.41 | -0.17 | *** |
| **Androgyny** |  |  |  |  |  |  |
| Androgyny t score | 0.09 | *** | 0.16 | 0.14 | 0.08 | *** |
| Androgyny diff score | 0.13 | *** | 0.18 | 0.09 | 0.12 | *** |

Correlation (Pearson).

Abbreviations: FEM(+)=Feminine personality traits (desirable); FEM(-)=Feminine personality traits (undesirable); MAS(+)= Masculine personality traits (desirable); MAS(-) =Masculine personality traits (undesirable); Androgyny t score=t statistic ratios of masculinity vs. femininity; Androgyny diff score=difference between masculinity score and femininity score; *** <0.05.

† n=1112.

‡ n=91.

§ n=1021.
