# Supplementary material for: Depression in relation to sex and gender expression among Swedish septuagenarians—Results from the H70 study
Source: PLoS One. 2020 Sep 14;15(9):e0238701. doi: 10.1371/journal.pone.0238701 (PMC7489509; doi:10.1371/journal.pone.0238701)
Supplement: S3 Table — Correlation (Pearson). Abbreviations: FEM(+) = Feminine personality traits (desirable); FEM(-) = Feminine personality traits (undesirable); MAS(+) = Masculine personality traits (desirable); MAS(-) = Masculine personality traits (undesirable). (DOCX) [file pone.0238701.s003.docx]

**S3 Table.**

| **Supplementary Table S3. Correlations between MADRS score and individual PN-SRI items** | | | |
| --- | --- | --- | --- |
|  |  |  |  |
|  | **N** | **Coefficient** | ***p*** |
| **FEM+** |  |  |  |
| Emotional | 1119 | 0.031 | *0.303* |
| Empathic | 1119 | 0.050 | *0.094* |
| Loving | 1118 | -0.054 | *0.070* |
| Passionate | 1119 | -0.007 | *0.826* |
| Sensitive | 1121 | 0.14 | ***** |
| Tender | 1116 | 0.012 | *0.697* |
| **FEM-** |  |  |  |
| Anxious | 1119 | 0.261 | ***** |
| Disoriented | 1119 | 0.191 | ***** |
| Naive | 1119 | 0.085 | ***** |
| Overcautious | 1120 | 0.097 | ***** |
| Oversensitive | 1118 | 0.208 | ***** |
| Self-doubting | 1119 | 0.171 | ***** |
| **MAS+** |  |  |  |
| Analytical | 1118 | -0.050 | *0.098* |
| Logical | 1121 | -0.061 | ***** |
| Objective | 1116 | -0.096 | ***** |
| Practical | 1121 | -0.115 | ***** |
| Rational | 1121 | -0.104 | ***** |
| Solution-focused | 1121 | -0.089 | ***** |
| **MAS-** |  |  |  |
| Arrogant | 1119 | 0.026 | *0.384* |
| Boastful | 1118 | 0.019 | *0.520* |
| Harsh | 1118 | 0.013 | *0.664* |
| Inconsiderate | 1118 | 0.063 | ***** |
| Ostentatious | 1118 | 0.055 | *0.066* |
| Power-hungry | 1118 | 0.040 | *0.183* |

Correlation (Pearson). Abbreviations: FEM(+)=Feminine personality traits (desirable); FEM(-)=Feminine personality traits (undesirable); MAS(+)= Masculine personality traits (desirable); MAS(-) =Masculine personality traits (undesirable)
