# Supplementary material for: Depression in relation to sex and gender expression among Swedish septuagenarians—Results from the H70 study
Source: PLoS One. 2020 Sep 14;15(9):e0238701. doi: 10.1371/journal.pone.0238701 (PMC7489509; doi:10.1371/journal.pone.0238701)
Supplement: S4 Table — Association between sex and a) depression status and b) MADRS score (step 1), adding gender expression as covariate (step 2) and as potential effect modifier (step 3). 1 Logistic regression. 2 Linear regression. Step 1 = Association between sex and depression (unadjusted model); Step 2 = Association between sex and depression, adding gender expression as covariate; Step 3 = Gender expression was tested as a potential effect modifier on the association between sex, any depression, and MADRS score (sex*gender expression). Abbreviations: MADRS score = burden of depressive symptoms; FEM(+) = Feminine personality traits (desirable); FEM(-) = Feminine personality traits (undesirable); MAS(+) = Masculine personality traits (desirable); MAS(-) = Masculine personality traits (undesirable); Androgyny t score = t statistic ratios of masculinity vs. femininity; Androgyny diff score = difference between masculinity score and femininity score; SE = Standard Error; *** <0.05. (DOCX) [file pone.0238701.s004.docx]

**S4 Table.**

**Table heading: Association between sex and a) depression status and b) MADRS score (step ^1^), adding gender expression as covariate (step ^2^) and as potential effect modifier (step ^3^)**

|  |  | **(a) Any depression**^†^ | | | | | **(b) MADRS score**^‡^ | | | | |
| --- | --- | --- | --- | --- | --- | --- | --- | --- | --- | --- | --- |
|  |  | **OR** | **B** | **SE** | **p** | **95% CI** | **R^2^** | **B** | **SE** | **p** | **95% CI** |
|  |  |  |  |  |  |  |  |  |  |  |  |
| **Unadjusted** |  |  |  |  |  |  |  |  |  |  |  |
|  | **Step ^1^** |  |  |  |  |  |  |  |  |  |  |
|  | Sex | 1.75 | 0.56 | 0.23 | *** | 1.12–2.75 | 0.005 | 0.78 | 0.30 | *** | 0.20–1.37 |
| **Femininity score** |  |  |  |  |  |  |  |  |  |  |  |
|  | **Step ^2^** |  |  |  |  |  |  |  |  |  |  |
|  | Sex | 1.47 | 0.38 | 0.24 | 0.11 | 0.92–2.33 | 0.041 | 0.33 | 0.30 | 0.27 | -0.25–0.92 |
|  | Femininity score | 1.04 | 0.04 | 0.01 | *** | 1.02–1.07 | 0.041 | 0.11 | 0.02 | *** | 0.08–0.14 |
|  | **Step ^3^** |  |  |  |  |  |  |  |  |  |  |
|  | Sex | 3.24 | 1.18 | 1.31 | 0.37 | 0.25–42.6 | 0.040 | 0.43 | 1.60 | 0.79 | -2.71–3.56 |
|  | Femininity score | 1.07 | 0.07 | 0.05 | 0.12 | 0.98–1.17 | 0.040 | 0.11 | 0.05 | *** | 0.004–0.22 |
|  | Sex*Femininity score | 0.98 | -0.02 | 0.03 | 0.54 | 0.94–1.04 | 0.040 | -0.002 | 0.03 | 0.95 | -0.07–0.06 |
| **FEM+ score** |  |  |  |  |  |  |  |  |  |  |  |
|  | **Step ^2^** |  |  |  |  |  |  |  |  |  |  |
|  | Sex | 1.62 | 0.48 | 0.24 | *** | 1.02–2.58 | 0.005 | 0.73 | 0.31 | *** | 0.13–1.33 |
|  | FEM+ score | 1.03 | 0.03 | 0.02 | 0.17 | 0.99–1.07 | 0.005 | 0.02 | 0.03 | 0.46 | -0.03–0.08 |
|  | **Step ^3^** |  |  |  |  |  |  |  |  |  |  |
|  | Sex | 1.39 | 0.33 | 1.33 | 0.80 | 0.10–19.0 | 0.004 | -0.02 | 1.69 | 0.99 | -3.33–3.29 |
|  | FEM+ score | 1.02 | 0.02 | 0.07 | 0.77 | 0.89–1.18 | 0.004 | -0.02 | 0.09 | 0.84 | -0.19–0.16 |
|  | Sex*FEM+ score | 1.01 | 0.01 | 0.04 | 0.91 | 0.92–1.10 | 0.004 | 0.025 | 0.06 | 0.65 | -0.08–0.14 |
| **FEM- score** |  |  |  |  |  |  |  |  |  |  |  |
|  | **Step ^2^** |  |  |  |  |  |  |  |  |  |  |
|  | Sex | 1.57 | 0.45 | 0.23 | 0.05 | 1.00–2.48 | 0.070 | 0.47 | 0.29 | 0.11 | -0.10–1.04 |
|  | FEM- score | 1.07 | 0.07 | 0.02 | *** | 1.03–1.10 | 0.070 | 0.21 | 0.02 | *** | 0.16–0.25 |
|  | **Step ^3^** |  |  |  |  |  |  |  |  |  |  |
|  | Sex | 3.51 | 1.25 | 0.78 | 0.11 | 0.76–16.1 | 0.070 | 1.39 | 0.88 | 0.12 | -0.35–3.12 |
|  | FEM- score | 1.14 | 0.14 | 0.07 | *** | 1.01–1.30 | 0.070 | 0.29 | 0.08 | *** | 0.13–0.45 |
|  | Sex*FEM- score | 0.96 | -0.04 | 0.04 | 0.27 | 0.89–1.03 | 0.070 | -0.05 | 0.05 | 0.27 | -0.15–0.41 |
| **Masculinity score** |  |  |  |  |  |  |  |  |  |  |  |
|  | **Step ^2^** |  |  |  |  |  |  |  |  |  |  |
|  | Sex | 1.69 | 0.53 | 0.23 | *** | 1.07–2.67 | 0.007 | 0.70 | 0.30 | *** | 0.11–1.29 |
|  | Masculinity score | 0.99 | -0.01 | 0.01 | 0.37 | 0.96–1.02 | 0.007 | -0.03 | 0.02 | 0.13 | -0.07–0.01 |
|  | **Step ^3^** |  |  |  |  |  |  |  |  |  |  |
|  | Sex | 0.56 | -0.57 | 1.28 | 0.65 | 0.05–6.90 | 0.006 | 0.87 | 1.72 | 0.62 | -2.51–4.24 |
|  | Masculinity score | 0.95 | -0.05 | 0.05 | 0.27 | 0.86–1.04 | 0.006 | -0.02 | 0.06 | 0.70 | -0.14–0.10 |
|  | Sex*Masculinity score | 1.03 | 0.03 | 0.03 | 0.38 | 0.97–1.09 | 0.006 | -0.004 | 0.04 | 0.92 | -0.08–0.07 |
| **MAS+ score** |  |  |  |  |  |  |  |  |  |  |  |
|  | **Step ^2^** |  |  |  |  |  |  |  |  |  |  |
|  | Sex | 1.69 | 0.52 | 0.23 | *** | 1.07–2.65 | 0.021 | 0.65 | 0.30 | *** | 0.07–1.23 |
|  | MAS+ score | 0.96 | -0.04 | 0.02 | 0.06 | 0.93–1.00 | 0.021 | -0.12 | 0.03 | *** | -0.17– -0.06 |
|  | **Step ^3^** |  |  |  |  |  |  |  |  |  |  |
|  | Sex | 0.79 | -0.23 | 1.23 | 0.85 | 0.07–8.89 | 0.020 | 0.52 | 1.74 | 0.76 | -2.88–3.93 |
|  | MAS+ score | 0.93 | -0.08 | 0.07 | 0.25 | 0.81–1.06 | 0.020 | -0.12 | 0.09 | 0.16 | -0.30–0.05 |
|  | Sex*MAS+ score | 1.03 | 0.03 | 0.04 | 0.53 | 0.95–1.11 | 0.020 | 0.004 | 0.06 | 0.94 | -0.10–0.11 |
| **MAS- score** |  |  |  |  |  |  |  |  |  |  |  |
|  | **Step ^2^** |  |  |  |  |  |  |  |  |  |  |
|  | Sex | 1.79 | 0.58 | 0.23 | *** | 1.14–2.83 | 0.009 | 0.89 | 0.30 | *** | 0.30–1.48 |
|  | MAS- score | 1.01 | 0.01 | 0.02 | 0.53 | 0.97–1.06 | 0.009 | 0.06 | 0.03 | *** | 0.01–0.12 |
|  | **Step ^3^** |  |  |  |  |  |  |  |  |  |  |
|  | Sex | 1.23 | 0.21 | 0.63 | 0.74 | 0.36–4.25 | 0.008 | 1.03 | 0.81 | 0.21 | -0.56–2.63 |
|  | MAS- score | 0.97 | -0.03 | 0.08 | 0.67 | 0.83–1.12 | 0.008 | 0.08 | 0.09 | 0.39 | -0.10–0.26 |
|  | Sex*MAS- score | 1.03 | 0.03 | 0.04 | 0.53 | 0.94–1.12 | 0.008 | -0.01 | 0.06 | 0.85 | -0.13–0.10 |
| **Androgyny t score** |  |  |  |  |  |  |  |  |  |  |  |
|  | **Step ^2^** |  |  |  |  |  |  |  |  |  |  |
|  | Sex | 1.70 | 0.53 | 0.23 | *** | 1.08–2.67 | 0.022 | 0.66 | 0.30 | *** | 0.08–1.24 |
|  | Androgyny t score | 1.18 | 0.16 | 0.10 | 0.11 | 0.96–1.44 | 0.022 | 0.70 | 0.16 | *** | 0.39–1.00 |
|  | **Step ^3^** |  |  |  |  |  |  |  |  |  |  |
|  | Sex | 1.48 | 0.39 | 0.35 | 0.27 | 0.74–2.96 | 0.023 | 0.26 | 0.45 | 0.56 | -0.62–1.15 |
|  | Androgyny t score | 0.96 | -0.05 | 0.43 | 0.92 | 0.42–2.20 | 0.023 | 0.09 | 0.54 | 0.87 | -0.97–1.15 |
|  | Sex*Androgyny t score | 1.13 | 0.12 | 0.24 | 0.61 | 0.71–1.79 | 0.023 | 0.38 | 0.32 | 0.24 | -0.25–1.00 |
| **Androgyny diff score** |  |  |  |  |  |  |  |  |  |  |  |
|  | **Step ^2^** |  |  |  |  |  |  |  |  |  |  |
|  | Sex | 1.68 | 0.52 | 0.23 | *** | 1.07–2.65 | 0.015 | 0.65 | 0.30 | *** | 0.07–1.23 |
|  | Androgyny diff score | 1.02 | 0.02 | 0.01 | 0.14 | 0.99–1.05 | 0.015 | 0.07 | 0.02 | *** | 0.03–0.12 |
|  | **Step ^3^** |  |  |  |  |  |  |  |  |  |  |
|  | Sex | 1.44 | 0.36 | 0.39 | 0.35 | 0.68–3.06 | 0.016 | 0.13 | 0.49 | 0.78 | -0.83–1.09 |
|  | Androgyny diff score | 0.99 | -0.01 | 0.06 | 0.90 | 0.88–1.12 | 0.016 | -0.02 | 0.08 | 0.77 | -0.17–0.13 |
|  | Sex*Androgyny diff score | 1.02 | 0.02 | 0.03 | 0.62 | 0.95–1.09 | 0.016 | 0.06 | 0.04 | 0.18 | -0.03–0.15 |

† Logistic regression.

‡ Linear regression.

**Step ^1^**= Association between sex and depression (unadjusted model); **Step ^2^**=Association between sex and depression, adding gender expression as covariate; **Step ^3^**= Gender expression was tested as a potential effect modifier on the association between sex, any depression, and MADRS score (sex*gender expression).

Abbreviations: MADRS score=burden of depressive symptoms; FEM(+)=Feminine personality traits (desirable); FEM(-)=Feminine personality traits (undesirable); MAS(+)= Masculine personality traits (desirable); MAS(-)=Masculine personality traits (undesirable); Androgyny t score=t statistic ratios of masculinity vs. femininity; Androgyny diff score=difference between masculinity score and femininity score; SE=Standard Error; *** <0.05.
