# Supplementary material for: Depression in relation to sex and gender expression among Swedish septuagenarians—Results from the H70 study
Source: PLoS One. 2020 Sep 14;15(9):e0238701. doi: 10.1371/journal.pone.0238701 (PMC7489509; doi:10.1371/journal.pone.0238701)
Supplement: S5 Table — Associations between gender expression and a) depression status and b) MADRS score by sex (complete Table 4). 1 Logistic regression. 2 Linear regression. Abbreviations: MADRS = Montgomery Åsberg Depression Rating Scale; FEM(+) = Feminine personality traits (desirable); FEM(-) = Feminine personality traits (undesirable); MAS(+) = Masculine personality traits (desirable); MAS(-) = Masculine personality traits (undesirable); Androgyny t score = t statistic ratios of masculinity vs. femininity; Androgyny diff score = difference between masculinity score and femininity score; OR = odds ratio; SE = Standard Error; *** <0.05. Model 1 (unadjusted); Model 3 (fully adjusted). aAdjusted for sex, living alone, having partner, partner loss, financial situation (making ends meet), and self-rated health. bAdjusted for sex, living alone, happy marriage, financial situation (making ends meet), and having confidant. cAdjusted for sex, living alone, having partner, happy marriage, having children, financial situation (making ends meet), and self-rated health. dAdjusted for sex, living alone, having partner, financial situation (making ends meet), and self-rated health. eAdjusted for sex, living alone, having partner, happy marriage, financial situation (making ends meet), and self-rated health. f Adjusted for financial situation (making ends meet). gAdjusted for sex, financial situation (making ends meet), and self-rated health. (DOCX) [file pone.0238701.s005.docx]

**S5 Table.** **Associations between gender expression and a) depression status and b) MADRS score by sex**

|  | **(a) Any depression^1^** | | | | | | | | | | **(b) MADRS score^2^** | | | | | | | | | |
| --- | --- | --- | --- | --- | --- | --- | --- | --- | --- | --- | --- | --- | --- | --- | --- | --- | --- | --- | --- | --- |
|  | **Model 1** | | | | | **Model 3** | | | | | **Model 1** | | | | | **Model 3** | | | | |
|  |  | | | | |  | | | | |  | | | | |  | | | | |
| **Women** |  | | | | |  | | | | |  | | | | |  | | | | |
|  | **OR** | **B** | **SE** | **p** | **95% CI** | **OR** | **B** | **SE** | **p** | **95% CI** | **R^2^** | **B** | **SE** | **p** | **95% CI** | **R^2^** | **B** | **SE** | **p** | **95% CI** |
| **Femininity** |  |  |  |  |  |  |  |  |  |  |  |  |  |  |  |  |  |  |  |  |
| Femininity score | 1.04 | 0.04 | 0.02 | *** | 1.01–1.07 | 1.02^a^ | 0.02 | 0.02 | 0.15 | 0.99–1.06 | 0.033 | 0.11 | 0.02 | *** | 0.06–0.15 | 0.107^a^ | 0.08 | 0.02 | *** | 0.03–0.13 |
| FEM+ score | 1.03 | 0.03 | 0.03 | 0.25 | 0.98–1.09 | 1.05^b^ | 0.04 | 0.03 | 0.15 | 0.99–1.10 | 0.001 | 0.03 | 0.04 | 0.43 | -0.05–0.12 | 0.06^b^ | 0.06 | 0.04 | 0.16 | -0.02–0.14 |
| FEM- score | 1.05 | 0.05 | 0.02 | *** | 1.01–1.10 | 1.03^c^ | 0.03 | 0.02 | 0.26 | 0.98–1.07 | 0.05 | 0.19 | 0.03 | *** | 0.12–0.25 | 0.116^c^ | 0.14 | 0.03 | *** | 0.08–0.20 |
| **Masculinity** |  |  |  |  |  |  |  |  |  |  |  |  |  |  |  |  |  |  |  |  |
| Masculinity score | 1.00 | -0.003 | 0.02 | 0.88 | 0.96–1.03 | 1.00^d^ | 0.004 | 0.02 | 0.84 | 0.97–1.04 | 0.002 | -0.03 | 0.03 | 0.29 | -0.09–0.03 | 0.078^d^ | -0.02 | 0.03 | 0.54 | -0.07–0.04 |
| MAS+ score | 0.97 | -0.03 | 0.03 | 0.29 | 0.93–1.02 | 0.99^e^ | -0.01 | 0.03 | 0.68 | 0.94–1.04 | 0.013 | -0.12 | 0.04 | *** | -0.20–-0.03 | 0.092^e^ | -0.08 | 0.04 | 0.06 | -0.15–0.01 |
| MAS- score | 1.02 | 0.02 | 0.03 | 0.37 | 0.97–1.08 | 1.02^f^ | 0.02 | 0.03 | 0.40 | 0.97–1.08 | 0.003 | 0.06 | 0.04 | 0.19 | -0.03–0.14 | 0.003^f^ | 0.06 | 0.04 | 0.19 | -0.03–0.14 |
| **Androgyny** |  |  |  |  |  |  |  |  |  |  |  |  |  |  |  |  |  |  |  |  |
| Androgyny t score | 1.22 | 0.20 | 0.12 | 0.10 | 0.96–1.54 | 1.11^g^ | 0.05 | 0.13 | 0.69 | 0.82–1.36 | 0.03 | 0.84 | 0.21 | *** | 0.43–1.25 | 0.092^g^ | 0.56 | 0.21 | *** | 0.15–0.98 |
| Androgyny diff score | 1.03 | 0.03 | 0.02 | 0.12 | 0.99–1.06 | 1.01^g^ | 0.01 | 0.02 | 0.75 | 0.97–1.04 | 0.02 | 0.10 | 0.03 | *** | 0.04–0.15 | 0.087^g^ | 0.06 | 0.03 | *** | 0.02–0.13 |
|  |  |  |  |  |  |  |  |  |  |  |  |  |  |  |  |  |  |  |  |  |
| **Men** |  |  |  |  |  |  |  |  |  |  |  |  |  |  |  |  |  |  |  |  |
|  | **OR** | **B** | **SE** | **p** | **95% CI** | **OR** | **B** | **SE** | **p** | **95% CI** | **R^2^** | **B** | **SE** | **p** | **95% CI** | **R^2^** | **B** | **SE** | **p** | **95% CI** |
| **Femininity** |  |  |  |  |  |  |  |  |  |  |  |  |  |  |  |  |  |  |  |  |
| Femininity score | 1.10 | 0.05 | 0.02 | *** | 1.01–1.10 | 1.05 ^a^ | 0.05 | 0.02 | *** | 1.01–1.10 | 0.043 | 0.11 | 0.02 | *** | 0.06–0.15 | 0.185^a^ | 0.10 | 0.02 | *** | 0.06–0.14 |
| FEM+ score | 1.03 | 0.03 | 0.03 | 0.44 | 0.96–1.10 | 1.05 ^b^ | 0.05 | 0.04 | 0.18 | 0.98–1.13 | 0.000 | 0.01 | 0.04 | 0.82 | -0.06–0.08 | 0.088^b^ | 0.03 | 0.04 | 0.39 | -0.04–0.10 |
| FEM- score | 1.10 | 0.09 | 0.03 | *** | 1.03–1.17 | 1.09 ^c^ | 0.08 | 0.04 | *** | 1.02–1.17 | 0.09 | 0.24 | 0.03 | *** | 0.17–0.30 | 0.223^c^ | 0.20 | 0.03 | *** | 0.13–0.26 |
| **Masculinity** |  |  |  |  |  |  |  |  |  |  |  |  |  |  |  |  |  |  |  |  |
| Masculinity score | 0.97 | -0.03 | 0.02 | 0.21 | 0.93–1.02 | 0.99 ^d^ | -0.01 | 0.02 | 0.56 | 0.94–1.03 | 0.002 | -0.03 | 0.02 | 0.27 | -0.08–0.02 | 0.152^d^ | -0.05 | 0.02 | 0.84 | -0.04–0.05 |
| MAS+ score | 0.95 | -0.05 | 0.03 | 0.09 | 0.89–1.01 | 0.98 ^e^ | -0.02 | 0.03 | 0.59 | 0.92–1.05 | 0.022 | -0.12 | 0.04 | *** | -0.19–-0.05 | 0.165^e^ | -0.05 | 0.03 | 0.12 | -0.12–0.02 |
| MAS- score | 1.01 | 0.01 | 0.04 | 0.88 | 0.94–1.08 | 1.01 ^f^ | 0.01 | 0.04 | 0.86 | 0.94–1.08 | 0.006 | 0.07 | 0.04 | 0.07 | -0.01–0.14 | 0.006^f^ | 0.07 | 0.04 | 0.07 | -0.01–0.14 |
| **Androgyny** |  |  |  |  |  |  |  |  |  |  |  |  |  |  |  |  |  |  |  |  |
| Androgyny t score | 1.08 | 0.08 | 0.20 | 0.71 | 0.72–1.61 | 0.89 ^g^ | -0.12 | 0.23 | 0.61 | 0.57–1.39 | 0.008 | 0.46 | 0.23 | *** | 0.01–0.91 | 0.141^g^ | 0.19 | 0.22 | 0.38 | -0.24–0.62 |
| Androgyny diff score | 1.01 | 0.01 | 0.03 | 0.77 | 0.95–1.07 | 0.99 ^g^ | -0.01 | 0.03 | 0.69 | 0.93–1.05 | 0.003 | 0.04 | 0.03 | 0.25 | -0.03–0.10 | 0.140^g^ | 0.01 | 0.03 | 0.74 | -0.05–0.07 |

^1^ Logistic regression. ^2^ Linear regression. Abbreviations: MADRS = Montgomery Åsberg Depression Rating Scale; FEM(+)=Feminine personality traits (desirable); FEM(-)=Feminine personality traits (undesirable); MAS(+)= Masculine personality traits (desirable); MAS(-)=Masculine personality traits (undesirable); Androgyny t score=t statistic ratios of masculinity vs. femininity; Androgyny diff score=difference between masculinity score and femininity score; OR=odds ratio; SE=Standard Error; *** <0.05.

**Model 1** (unadjusted); **Model 3** (fully adjusted).

^a^Adjusted for sex, living alone, having partner, partner loss, financial situation (making ends meet), and self-rated health. ^b^Adjusted for sex, living alone, happy marriage, financial situation (making ends meet), and having confidant. ^c^Adjusted for sex, living alone, having partner, happy marriage, having children, financial situation (making ends meet), and self-rated health. ^d^Adjusted for sex, living alone, having partner, financial situation (making ends meet), and self-rated health. ^e^Adjusted for sex, living alone, having partner, happy marriage, financial situation (making ends meet), and self-rated health. ^f^ Adjusted for financial situation (making ends meet). ^g^Adjusted for sex, financial situation (making ends meet), and self-rated health.
