# Supplementary material for: Depression in relation to sex and gender expression among Swedish septuagenarians—Results from the H70 study
Source: PLoS One. 2020 Sep 14;15(9):e0238701. doi: 10.1371/journal.pone.0238701 (PMC7489509; doi:10.1371/journal.pone.0238701)
Supplement: S1 Text — (DOCX) [file pone.0238701.s006.docx]

**S6 Text.**

When excluding participants with depression in the third sensitivity analysis, some associations between gender expression and MADRS score were modified. For Models 1-3 (Table 2 (b)), the associations for total femininity score, FEM+, MAS-, androgyny t score, and androgyny difference score were supported. The results changed for total masculinity score in Model 1 (R^2^=0.004; B=-0.03; SE=0.01; *p*<0.05) and Model 2 (R^2^=0.03; B=-0.03; SE=0.01; *p*<0.05), MAS- in Models 2 and 3 (R^2^=0.001; B=0.02; SE=0.02; *p*=0.23), and FEM+ in Model 3 (R^2^=0.011; B=0.01; SE=0.02; *p*=0.48). For both sexes (Table 3(b)), the associations for total femininity score, FEM+, FEM-, total masculinity score, MAS-, androgyny t score, and androgyny difference score were supported. The results for MAS+ changed for women in Model 1 (R^2^=0.003; B=-0.04; SE=0.03; *p*=0.09) and for men in Model 3 (R^2^=0.078; B=-0.08; SE=0.02; *p*<0.05).
